# Unusual case reports: Tardive oculogyric crisis (tardive syndromes)

**DOI:** 10.4103/0019-5545.37326

**Published:** 2007

**Authors:** V. N. Vahia, P. M. Naik, P. Deotale

**Affiliations:** Department of Psychiatry, Dr. R. N. Cooper Hospital (Temporarily relocated at V. N. Desai Hospital), V. N. Desai Road, Santacruz (E), Mumbai, Maharashtra, India

**Keywords:** Olanzapine, promethazine, psychotic symptoms, tardive oculogyric crisis

## Abstract

We report, we believe for the first time in India, two cases of tardive oculogyric crisis in patients of schizophrenia, paranoid subtype (DSM IV code 295.30) during olanzapine therapy. Relevant data on this rare condition is discussed.

Chiu[1] coined the term “tardive oculogyric crisis” to describe the clinical presentation of stereotype repetitive auditory hallucinations during acute oculogyric crisis, amongst patients receiving antipsychotic medication. Several authors equate this phenomenon with tardive dyskinesia as this cluster of symptoms occurs after several years of neuroleptic drug treatment.[2,3] Dave[4] used the term when the oculogyric crisis occurred late in treatment without exacerbation of psychotic symptoms.

## CASE REPORTS

### Case 1

A 35-year-old diagnosed case of schizophrenia, paranoid subtype (DSM IV code 295.30), of 15 years' duration, was receiving oral olanzapine (20 mg/day) for the past 30 months. She complained of recent onset intermittent up-rolling of eyeballs, associated with anxiety, dryness of mouth, giddiness and third-person auditory hallucinations of derogatory contents. The stereotyped cluster of symptoms occurred 8-10 times per day during the six to seven days before coming to the clinic. She could voluntarily bring down the eyes to normal position, for a few minutes, after which the eyes would roll up again. She was distressed by these symptoms and expressed passive suicidal ideas and death wishes if the symptoms were to persist. In the past she had experienced acute oculogyric crisis while she was being treated with oral trifluperazine, trihexyphenidyl, risperidone and carbamazepine. She was hospitalized. Repeated EEGs during the acute episodes did not reveal any abnormality. Her dose of olanzapine was reduced to 15 mg, and injection promethazine was administered whenever acute episodes of oculogyric crisis recurred. Both oculogyric crisis and associated stereotyped cluster of psychotic symptoms remitted. The diagnosis of neuroleptic induced tardive oculogyric crisis was considered. Oral promethazine (50 mg/day in two divided doses) was added. Frequency of the distressing episodes has reduced to one to two episodes per week. She is being helped to accept the symptoms and cope with them without getting impatient.

### Case 2

A 30-year-old diagnosed case of schizophrenia, paranoid subtype (DSM IV code 295.30) of five years' duration, complained of upward deviation of eyeballs, intermittent spasmodic deviation of neck and trunk, suspiciousness and hearing abusive voices inaudible to others. He was consuming unsupervised oral combination of haloperidol, trifluperazine and trihexyphenidyl for eight months. He was hospitalized for the aforementioned complaints and was treated initially with injection promethazine, along with oral trihexyphenidyl and clonazepam. After alleviation of the acute extrapyramidal symptoms attributed to the neuroleptic drugs, low dose (2.5 mg) olanzapine was administered orally and was gradually enhanced to 15 mg. About seven months later, he complained of recurrence of up-rolling of eyeballs, associated with agitation, restlessness, anxiety and increase in the intensity of auditory hallucinations. The initial frequency of such episodes was six to eight times a week. Oral promethazine (50 mg/day) was added, and the frequency of such episodes reduced to one or two episodes a week. The psychotic symptoms also came under control.

## DISCUSSION

Olanzapine is an atypical antipsychotic agent. It is reported to lack the propensity to cause tardive dyskinesia.[5] Recent reports suggest that olanzapine can improve tardive dyskinesia.[6,7] On the other hand, several other authors report occurrence of tardive dyskinesia or dystonia after prolonged use of olanzapine.[8,9] We report two patients who developed tardive oculogyric crisis with olanzapine. They responded to promethazine. Tardive oculogyric crisis merits vigilant detection as it can occur with atypical antipsychotic agents like olanzapine.

Acute oculogyric crisis in a patient on antipsychotic drug therapy is a familiar phenomenon. The presentation is a specific dystonia, and differential diagnosis is almost never considered. Recurrent oculogyric crisis is distinctly different from the acute adverse drug event. It has been variously considered to be a form of tardive dyskinesia,[2,3] recurrent acute dyskinesia or a conversion symptom.[10]

Recurrent exacerbation of psychotic symptoms associated with episodes of oculogyric crisis may prompt the clinician to enhance the dose of antipsychotic medication. This is not a recommended strategy, particularly since the remission of the oculogyric crisis with anticholinergic drug is associated with remission of psychotic symptoms as well. Discontinuing or tapering the dose of neuroleptic drug or switching to a drug with lesser risk of acute extrapyramidal side effect is recommended.

Choreo-athetoid tardive dyskinesia is a differential diagnosis in case of recurrent oculogyric crisis. The differentiating feature is that choreo-athetoid dyskinesia worsens on anticholinergic drug administration, dose tapering or omission of the drug or on switching over to another drug with lower adverse drug reaction risk.[10] Most patients of recurrent oculogyric crisis are aware of the attacks and find them unpleasant. Under strong suggestion or with voluntary efforts, the victim can bring down the up-rolled eyes for a brief period. However, the eye balls roll up again, either spontaneously or when the patient is emotionally aroused. Recurrence of the till-then-controlled psychotic symptoms accompanies the oculogyric symptoms. A baffled clinician may thus misdiagnose it as psychogenic or a hysterical event. Some clinicians refuse to recognize its occurrence at all.[10]

Troublesome recurrent acute oculogyric crisis is likely to respond to a switch to a low-potency neuroleptic drug, an atypical antipsychotic drug, tapered dose or enhanced inter-dose interval of the current drug.

One author has reported the spontaneous resolution of the stereotyped psychiatric symptoms when oculogyric crisis abated, in a series of 10 patients with at least five years of neuroleptic exposure.[10]

## References

[1] Chiu L (1989). Transient recurrences of auditory hallucinations during acute dystonia. Br J Psychiatry.

[2] Sachdev P (1993). Tardive and chronically recurrent oculogyric crisis. Mov Disord.

[3] Leigh R, Foley J, Remler B, Civil R (1987). Oculogyric crisis: a syndrome of thought disorder and ocular deviation. Ann Neurol.

[4] Dave M (1994). Tardive oculogyric crisis with clozapine. J Clin Psychiatry.

[5] Beasley CM, Dellva MA, Tamura RN, Morgenstern H, Glazer WM, Ferguson K (1999). Randomized double-blind comparison of the tardive dyskinesia in patients with schizophrenia during long-term treatment with olanzapine or haloperidol. Br J Psychiatry.

[6] Littrell KH, Johnson CG, Littrell S, Peabody CD (1998). Marked reduction of tardive dyskinesias with olanzapine. Arch Gen Psychiatry.

[7] Jaffe ME, Simpson GM (1999). Reduction of tardive dyskinesia with olanzapine. Am J Psychiatry.

[8] Ananth J, Kenan J (1999). Tardive dyskinesia associated with olanzapine monotherapy. J Clin Psychiatry.

[9] Dunayevich E, Strakowsky SM (1999). Olanzapine-induced tardive dystonia. Am J Psychiatry.

[10] Benjamin S (1987). Oculogyric crisis: Movement disorder in neurology and neuropsychiatry.

